# Co-simulation framework combining a microscopically detailed point neuron model of the hippocampal CA1 region with the macroscopic high-resolution virtual brain model

**DOI:** 10.1007/s10827-026-00925-w

**Published:** 2026-02-23

**Authors:** Lorenzo Tartarini, Paul Triebkorn, Lionel Kusch, Sergio Solinas, Huifang Wang, Daniela Gandolfi, Viktor Jirsa, Jonathan Mapelli

**Affiliations:** 1 Department of Biomedical, Metabolic and Neural Sciences, University of Modena and Reggio Emilia, Modena, Italy; 2 Aix Marseille Univ, INSERM, INS, Inst Neurosci Syst, Marseille, France; 3 Department of Engineering, University of Sassari, Sassari, Italy; 4 Department of Engineering “Enzo Ferrari”, University of Modena and Reggio Emilia, Modena, Italy; 5 Artificial Intelligence Research and Innovation Center AIRI, University of Modena and Reggio Emilia, Modena, Italy; 6 Center for Neuroscience and Neurotechnologies, University of Modena and Reggio Emilia, Modena, Italy

**Keywords:** Co-simulation, Whole brain models, Epileptor, Spiking neural networks

## Abstract

The human brain is a complex adaptive system characterized by dynamic processes operating across multiple spatio-temporal scales. Capturing these dynamics requires computational models that can integrate different levels of resolution. In this work we present a multiscale co-simulation framework that couples whole-brain modeling with a detailed point-neuron model of the human hippocampal CA1 region. We used a high-resolution implementation of the “The Virtual Brain” (TVB), in which cortical surface mesh vertices are embedded with the Spatial Epileptor Model (SEM). At the microscale, the CA1 model captures neuronal activity at micrometer spatial and sub-millisecond temporal resolution. This integration enables the simulation of macroscale epileptic dynamics with microscale neuronal precision within anatomically grounded brain regions, facilitating cross-scale communication. These results demonstrate the potential of this approach to advance mechanism-driven, personalized medicine in clinical neuroscience.

**Supplementary Information:**

The online version contains supplementary material available at 10.1007/s10827-026-00925-w.

## Introduction

Computational brain models have proven essential for advancing our understanding of brain functions and for developing innovative technologies to investigate neurological disorders and design pharmacological interventions. In recent years, research in this field has produced a wide range of models capable of replicating brain dynamics at multiple spatio-temporal scales. High-resolution models have been particularly effective in elucidating local brain dynamics (Gandolfi et al., 2023), whereas large scale models are increasingly adopted as digital twins for clinical applications (Wang et al., 2024). Despite extensive efforts and investments, most simulations at the cellular level remain limited to animal studies. Consequently, co-simulation technologies, where regions of interest are modeled at high resolution while the rest of the brain is approximated using mean field approaches, have thus been developed primarily in mouse models (Kusch et al., 2024). Only recently, alternative co-simulation frameworks have been proposed to model multiscale dynamics; however, beside interesting results with mouse models (Kusch et al., 2024; Hater et al., 2025), the only report at human-level simulations (Bragin et al., 2024) presents technical details about the interface between micro and macroscale simulators without simulation results.

The possibility of simultaneously simulating human brain dynamics at multiple scales would represent a major advance for both diagnosis and therapy. In the context of excitability disorders, such as epilepsy, this is particularly relevant. Epileptic seizures are marked by recurrent, spontaneous, and complex spatiotemporal activity that involves multiple brain regions and diverse propagation patterns, including frequency shifts, latency differences, and synchronization. Understanding how seizures evolve in space (i.e., which brain regions are involved) and time (i.e., the sequence and dynamics of activity spread) is critical for accurate diagnosis, seizure prediction, and for designing effective treatments, including surgical interventions. To this end, we present a novel co-simulation framework that integrates both macroscale and microscale levels of brain modeling, enabling the study of human brain dynamics with high spatial and temporal resolution.

**Fig. 1:**
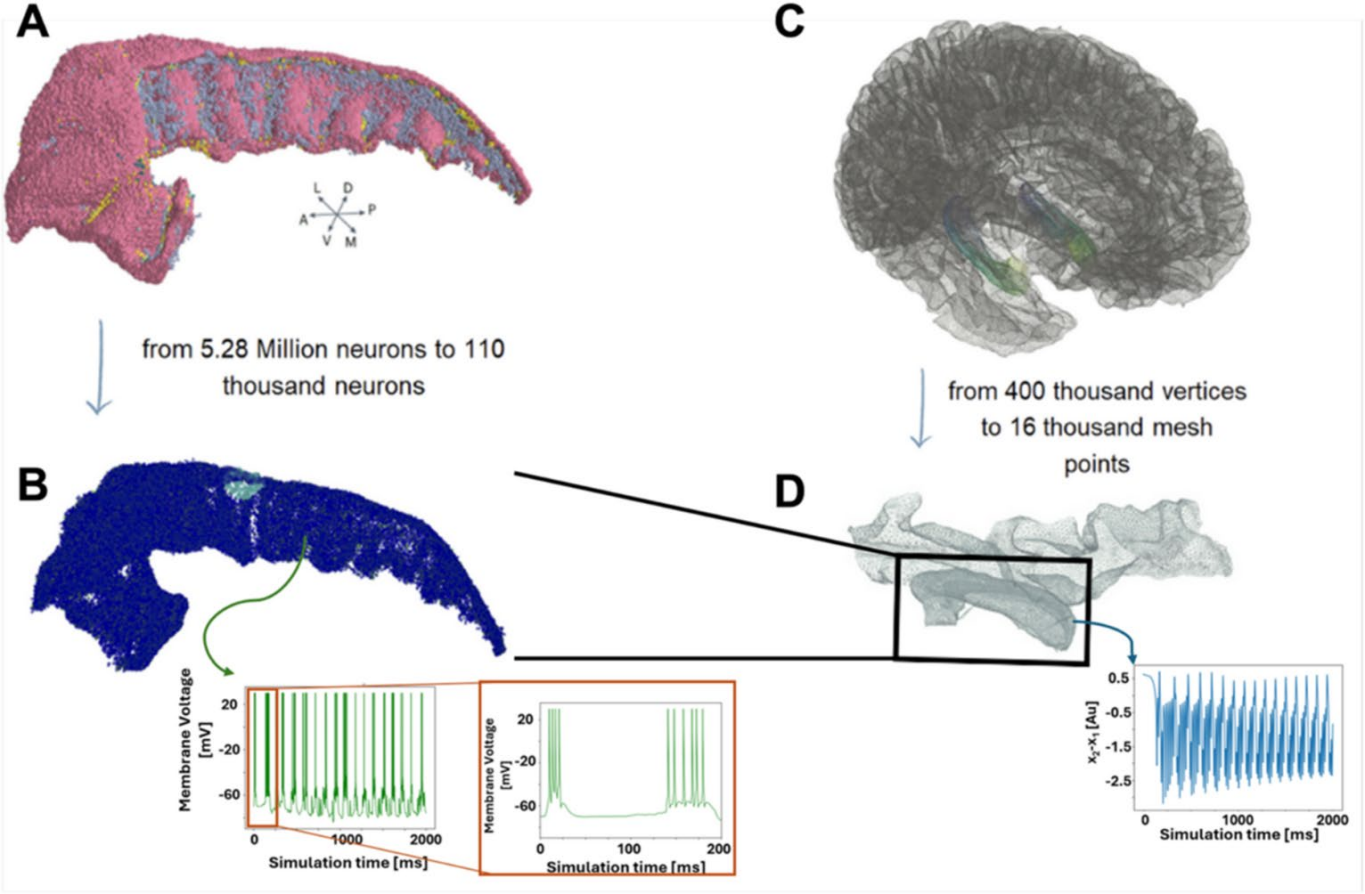
Different computational framework. **A**. Full-scale model of the CA1 region of a right hippocampus as obtained in Gandolfi et al. (2023). **B**. The full-scale model has been downscaled for computational efficiency in the testing phase. Insets show the typical bursting behavior of a CA1 neuron. **C**. The high-resolution TVB model was downscaled for computational efficiency. **D**. Parahippocampal and hippocampal regions of the TVB. Black box shows the region corresponding to the right hippocampus. Inset shows seizure-like activity represented by the difference between the slow waves state $x_2$ and the fast discharges state $x_1$

## Methods

### Co-simulation framework

In this work, we present a co-simulation framework that integrates a microscopically detailed spiking neural network model of the human CA1, simulated using the NEST (based on NEST 3.8.0-post0.dev0; see Supplementary materials) framework (Gewaltig & Diesmann, 2007; Graber et al., n.d.), with macroscopic whole-brain dynamics simulated using The Virtual Brain (TVB; tvb-library 2.3; See supplementary materials) platform (Sanz-Leon et al., 2015). The two main constraints of the framework are scalability and computational efficiency, both of which are essential to support increasing complexity in neural population modeling and whole brain simulations. To tackle the first aspect, the communication protocol transmits rate-coded information for each region in both simulations (see Fig. SM1). To ensure the computational efficiency, the system has been implemented in a containerized environment, enabling straightforward portability and execution across High Performance Computing (HPC) platforms, and has been validated on such systems.

### Hippocampus CA1 NEST model

The CA1 region of the human hippocampus was simulated in NEST as in Gandolfi et al. (2023). Briefly, the point-neuron model incorporates data-driven 3D soma positioning (Fig. 1) with the corresponding connectivity matrix generated through a realistic morpho-anatomical connection strategy (Gandolfi et al., 2022). Each neuron is simulated using the Hill-Tononi (HT) model (Hill & Tononi, 2005) which, although originally developed for thalamic neurons, can reproduce hippocampal bursting activity and provides a consistent number of degrees of freedom with a balanced compromise between computational efficiency and detailed dynamics. Moreover, HT neurons support intrinsic fast and slow excitatory and inhibitory input dynamics, enabling synaptic behavior consistent with AMPA, NMDA and GABAergic transmission. Synaptic communication between point neurons is governed by the Tsodyks-Markram model (Tsodyks & Markram, 1997). Such a model captures both the availability of synaptic resources and the probability of neurotransmitter release, enabling short term plasticity. Equations of the HT and Tsodyks-Markram models are detailed in the Supplementary Materials. The network dynamics are simulated at a sub-millisecond time and micrometer spatial resolution, necessitating the use of HPC resources for networks with billions of synapses.

To enable coupling between TVB-based simulation and the NEST-based model, custom input/output devices have been designed in NEST (Kusch et al., 2024) using the Message Passing Interface (MPI) protocol to receive and send data at every simulation time step. The NEST input device receives a rate-coded signal and generates Poissonian spike trains with the received firing rate.

An input device is created for each CA1 mesh vertex, with each device driving a corresponding subpopulation of point neurons, as described below. At each time step, a rate vector is received, containing one value per neuronal subpopulation. The order of the vectors is consistent with the order of creation of the input devices. The NEST MPI-based output device follows a similar design. It records the total number of spike events for each subpopulation and transmits a vector of spike counts back to the TVB process, enabling feedback-driven co-simulation. This will later be used as input for the TVB model. The input/output devices are compatible with multi-node parallel execution, allowing the framework to scale efficiently with increasing network size and complexity, a critical requirement given the growth in computational cost associated with large-scale spiking networks. In the presented study, a down-sampled version of the right CA1 network model was used. The original model, comprising about 5.28 million neurons and 40 billion synapses, was scaled down to 110 000 neurons and 4.3 million connections (Fig. 1B; see Methods). The downscaling procedure has been performed by randomly sampling neurons and, accordingly, preserving only consistent connections. The integrity of the reduced network has been assessed by analyzing the neuronal density distribution (Fig. SM3 A-C), the indegree and outdegree distribution (Fig. SM3 D-E) and the distribution of the connection lengths (Fig. SM3 F) (See Supplementary materials).

### The high-resolution virtual brain epileptor model

**Fig. 2:**
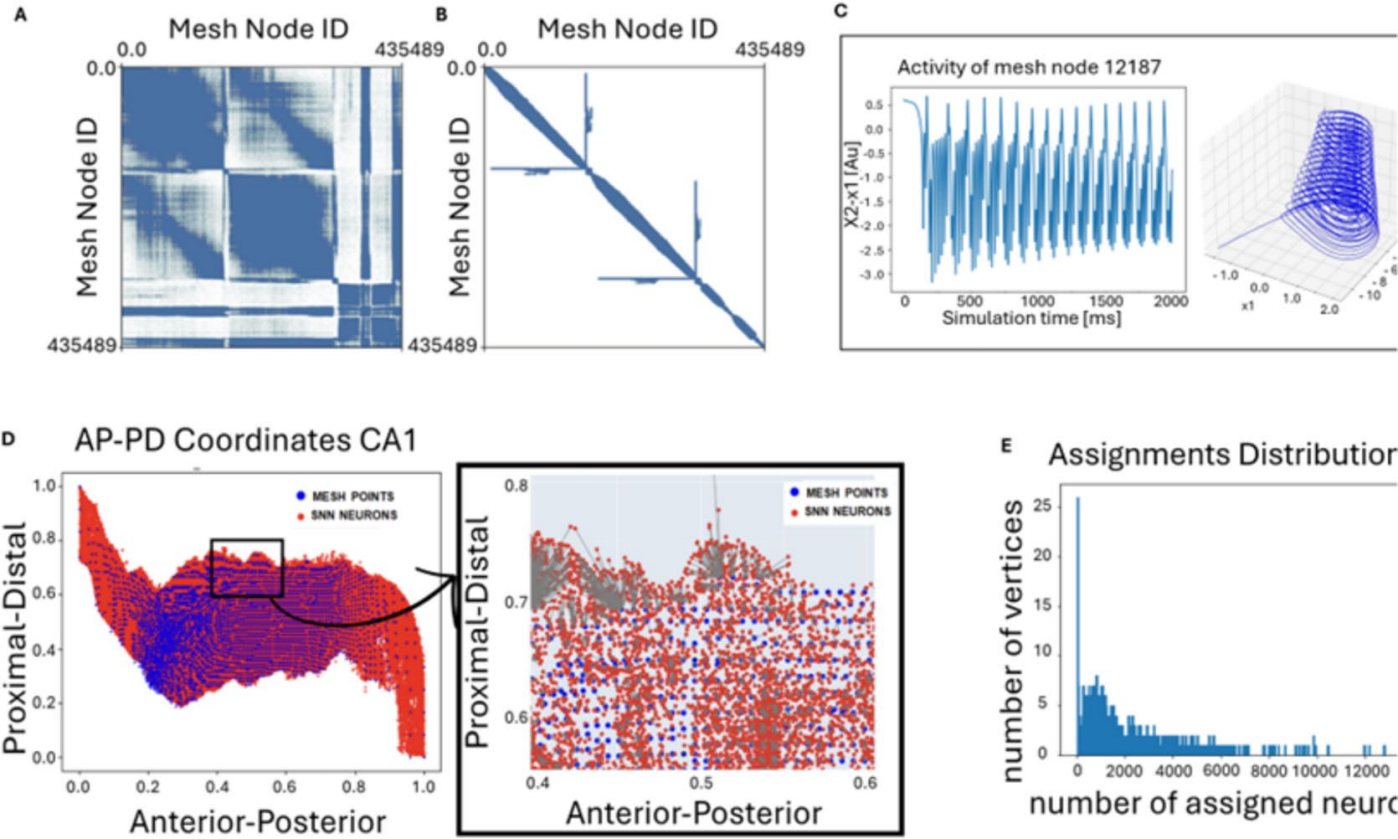
High Resolution version of TVB, using subject’s mesh points as nodes. (**A**) Global Connectivity Matrix obtained from Tractography, (**B**) Local Connectivity based on Geodesic distance between the nodes. (**C**) SEM activity ($x_2-x_1$) timeseries of a single TVB node and corresponding $x_1$, $y_1$ subpopulation dynamic driven by permittivity state *z* reproducing seizure like oscillations. (**D**) Assignment between TVB mesh nodes and NEST model neurons using the HippUnfold protocol (DeKraker et al., 2022, 2023) to bring the two sets of points in a common coordinate system. (**E**) Histogram showing the number of vertices in the mesh assigned to a certain number of neurons

The macroscopic brain model in TVB was built using anatomical data derived from T1-weighted and diffusion-weighted magnetic resonance imaging (DW-MRI) (Wang et al., 2024) provided by the human connectome project (Van Essen et al., 2013; Glasser et al., 2013, subject 100206). From these imaging modalities, a cortical surface mesh was extracted (Fig. 1C) and parcellated using the Virtual Epileptic Patient atlas (Wang et al., 2021), generating a map between each mesh vertex and its corresponding brain region. Then, using DW-MRI tractography, a sparse connectivity matrix has been generated between the vertices of the surface mesh, where each weight is proportional to the cross-sectional area of the streamlines connecting the vertices (Triebkorn et al., 2025). Each node of the surface mesh, representing a neural mass of the TVB network, was simulated using the Spatial Epileptor Model (SEM) (Proix et al., 2018) which captures the onset, time course, and termination of ictal-like discharges, as well as their recurrence (Jirsa et al., 2014). Neural masses were coupled via two types of connections: (i) long-range global connectivity derived from tractography (Fig. 2A), and local connections (Fig. 2B), based on a Laplacian kernel spanning over the geodesic distance between nodes along the mesh surface. The resulting global connectivity exhibits a block-structured organization, revealing distinct inter-hemispheric, intra-hemispheric, and subcortical connections, as well as projections from subcortical to cortical regions. Due to the high dimensionality of the system, the visualizations of Fig. 2 A, B display only the sparsity pattern of the connectivity matrix, omitting connection strength to avoid visual clutter. Moreover, the projection of this inherently three-dimensional connectivity structure onto two-dimensional plane introduces artifacts, which stem from mesh node indexing and the high dimensionality of the dataset.

The 6D Spatial Epileptor Model is described by a system of 6 differential equations (see supplementary material) representing distinct dynamics states: two fast discharge variables ($x_1$, $y_1$), two spike and wave events (SWE) states ($x_2$, $y_2$), one permittivity state (*z*), and an integral coupling function (g). Specifically, $x_1$ models fast excitatory activity during seizures and $y_1$ acts as the associated recovery variable. The variables $x_2$ and $y_2$ represent slower oscillatory SWE activity and its corresponding recovery dynamics, respectively. The variable *z* modulates seizure susceptibility and governs transitions between ictal and non-ictal states. The integral coupling function g links the two subpopulations, driving the SWE states by integrating past fast discharge activity via temporal convolution. The external input currents to the rapid discharge population $I_{ext}$ and the SWE population $I_{ext2}$, are constant bias terms that dictate the stable points of the corresponding subsystems. The parameter m controls the dynamics of SEM during the ictal period, while the excitability parameter $x_0$ controls the equilibrium point of the SEM, thus the ability, for each node, to switch between a non-ictal and an ictal state (Fig. 2C). Moreover, the SEM includes global coupling applied to the variable $x_1$, local coupling influences both the dynamics of $x_1$ and $x_2$ and the integral coupling function g. The global coupling is based on a weighted global connectivity signal propagation, where a history buffer containing all neural masses’ previous states is sampled according to a delay matrix based on tract length between regions. Local coupling models the influence of the activity of a node on its neighbors by: (i) rapid discharge coupling ($loc_{11}$), (ii) an integral coupling term ($loc_{12}$), and (iii) the effects of coupling through SWE events ($loc_{22}$), where $loc_{11}$, $loc_{12}$, $loc_{22}$ are the corresponding local coupling strengths. In particular, the *g* state directly modulates the SWE activity, thus creating a bidirectional link between the fast discharge and the SWE states in neighboring regions. The whole brain mesh consists of 337,981 cortical nodes and 97,509 subcortical nodes, totaling 435,490 total locations. Since both global connectivity, tract length mapping and geodesic distances would result in extremely large and sparse matrices, custom TVB classes have been used. The custom SimulatorSparse class includes a specialized SparseHistory class attribute that keeps track of states for delayed communications. Local coupling is handled by the HeavisideSparse function, optimized via a Numba-accelerated sparse matrix routine. Global connectivity is represented by the ConnectivitySparse class, which stores weights, tract lengths, and delays using sparse matrices.

For validation, a reduced mesh was used, including only regions from the right hemisphere: fusiform gyrus, para-hippocampal gyrus, entorhinal cortex, subiculum, CA1, CA2, CA3, CA4 and dentate-gyrus, as extracted from MRI data. The excitability parameter ($x_0$) was set to model CA1 as the epileptogenic zone ($x_0$= -1.6), while the surrounding areas were defined as propagation zones ($x_0$ = -1.9). The system was initialized at a stable point ($x_1$ = -1.5, $y_1$ = -11, *z* = 3, $x_2$ = -0.9, $y_2$ = 0.3, *g* = -0.1), except for a localized subset of adjacent CA1 nodes, designated as the seizure onset zone, where the epileptic oscillations triggered pathological propagation ($x_1$ = 0, $y_1$ = -5, *z* = 3, $x_2$ = 0, $y_2$ = 0, *g* = 0). The local coupling parameters were calibrated to ensure coherent propagation of both dynamics $x_1$ and $x_2$ throughout the network with the following values (k = 0.636, $\gamma _{11}$ = 0.34, $\gamma _{12}$ = 0.064, $\gamma _{22}$ = 0.032, $\gamma _{glob}$ = 1, $\theta _{11}$ = -1, $\theta _{12}$ = -1, $\theta _{22}$ = -0.5, $I_{ext}$ = 3.1, $I_{ext2}$ = 0.45, $\tau _0$ = 2857, $\tau _1$ = 1, $\tau _2$ = 10, *tt* = 0.17)

### Epileptor activity to NEST rates conversion

The two simulators were coupled through MPI based communication protocol that enabled bidirectional exchange of activity across the two spatio-temporal scales. To have topologically coherent co-simulation across models, neurons were assigned to vertices of the TVB mesh by mapping each vertex to the spatially nearest NEST neurons. This mapping was performed using geodesic Laplace coordinates along the Anterior-Posterior (AP) and Proximal-Distal (PD) axes of the hippocampal surfaces, as computed by the HippUnfold toolbox (DeKraker et al., 2022, 2023). The HippUnfold toolbox provides a surface-based topology-preserving representation of the hippocampus based on thickness, curvature, gyrification, texture and other morphological and laminar features (DeKraker et al., 2022). This surface-based representation is better suited for anatomically meaningful alignment between subjects than conventional volumetric registrations (DeKraker et al., 2023).

After projecting both the 3D coordinates of the point neurons and the mesh vertices into a common 2D intrinsic coordinate system, a KDTree clustering algorithm was used to identify the closest neurons to each mesh vertex (Fig. 2D). The assignment was limited to mesh nodes labeled as CA1, CA2 or Subiculum. To refine anatomical labeling, mesh nodes originally labeled CA2 or subiculum but assigned to any point neuron were relabeled CA1. In contrast, mesh nodes initially labeled CA1 that remained unassigned were relabeled CA2 or subiculum, depending on the identity of the nearest non-CA1 mesh node.

The framework employs a Singularity container (Kurtzer et al., 2017) for portability across HPC systems and is organized into three main Python-based processes. The launcher process manages the sequential initialization of the two simulations. It first starts the TVB simulation, which generates the model, and then opens an MPI communication port via a custom model class. This port expects as many inputs/outputs as there are surface vertices labeled CA1, the region targeted for co-simulation. When the MPI port is active, the launcher starts the NEST simulation, which connects to the port using custom developed streaming I/O devices: spike_detector_mpi and spike_generator_mpi. For each CA1 mesh node, a pair of these devices is instantiated and connected to the corresponding neurons, based on the AP-PD mapping. Spike_generator_mpi receives input rates from the MPI port and generates the corresponding spike trains using an inhomogeneous Poisson process. These spikes are relayed to NEST point neuron models through parrot neurons and static synapses. In contrast, spike_detector_mpi is designed to transmit the number of spikes detected within each simulation time step back to the MPI port. However, in this preliminary version of the framework, this feedback is disabled. TVB influences CA1 spiking activity, but not vice versa.

To convert the absolute state variables of the SEM into meaningful spiking input, a Rectified Linear Unit (ReLU)-like transformation is applied to a combination of $x_2$ slow wave events and $x_1$ fast discharges (Eq. (1)-(3)). This produces a firing rate which, scaled by the simulation timestep, defines the Poisson parameter used to probabilistically generate spikes at each time step for each subpopulation.

1 $$\begin{aligned} p_{x_1}= (x_1 + o_{x_1})\cdot g_{x_1}\cdot dt \end{aligned}$$

2 $$\begin{aligned} p_{x_2}= (x_2 + o_{x_2})\cdot g_{x_2}\cdot dt \end{aligned}$$

3 $$\begin{aligned} spike\_generation = Poisson(p_{x_1} + p_{x_2}) \end{aligned}$$

Where $o_{x_1}$, $o_{x_2}$ are the conversion offsets and $g_{x_1}$, $g_{x_2}$ are the conversion gains. The conversion parameters were configured such that at least one NEST spike is generated for each SWE Complex, modeled through the $x_2$ state, within the connected subpopulation. Additionally, the probability of generating in response to the rapid discharge component ($x_1$) denoted as $p_{x_1}$, was set to be half the probability associated with the SWE complex ($p_{x_2}$). This parameterization results in Poisson-distributed firing rates ranging from 0 to 0.31 spikes/s for $x_2$-driven activity and from 0 to 0.16 spikes/s for $x_1$-driven activity. These values were derived using the following $o_{x_2}$= 0.5, $o_{x_1}$= 2, $g_{x_2}$= 1.25, $g_{x_1}$= 0.375 and a simulation step dt = 0.1 ms for both NEST and TVB simulations. This conversion scheme is grounded in the observation of spiking synchronization driven by slow SWE dynamics, as described in Truccolo et al. (2014), where neuronal firing showed temporal clustering at the onset of the spike phase during recorded seizures. A secondary weaver spiking activity was also observed during the wave phase, which in the present model is captured via the $x_1$ component, modulating the contribution of rapid discharges in the SEM-to-spike-rate transformation. At each simulation time step, the received values $p_{x_1}$ and $p_{x_2}$ drive a Poisson process that determines whether each subpopulation generates a spike (i.e., $spike\_generation$). To validate the co-simulation framework, an initial test was performed with SNN connectivity disabled, in order to confirm the synchronization between SEM-derived $x_1$ and $x_2$ activity and subpopulation firing rates. Subsequent simulations with NEST connectivity enabled the investigation of the dynamics of network-level signal propagation. Extensive tests were conducted to assess the correct interaction between TVB and NEST simulators (see Supplementary materials for a full description).

**Fig. 3:**
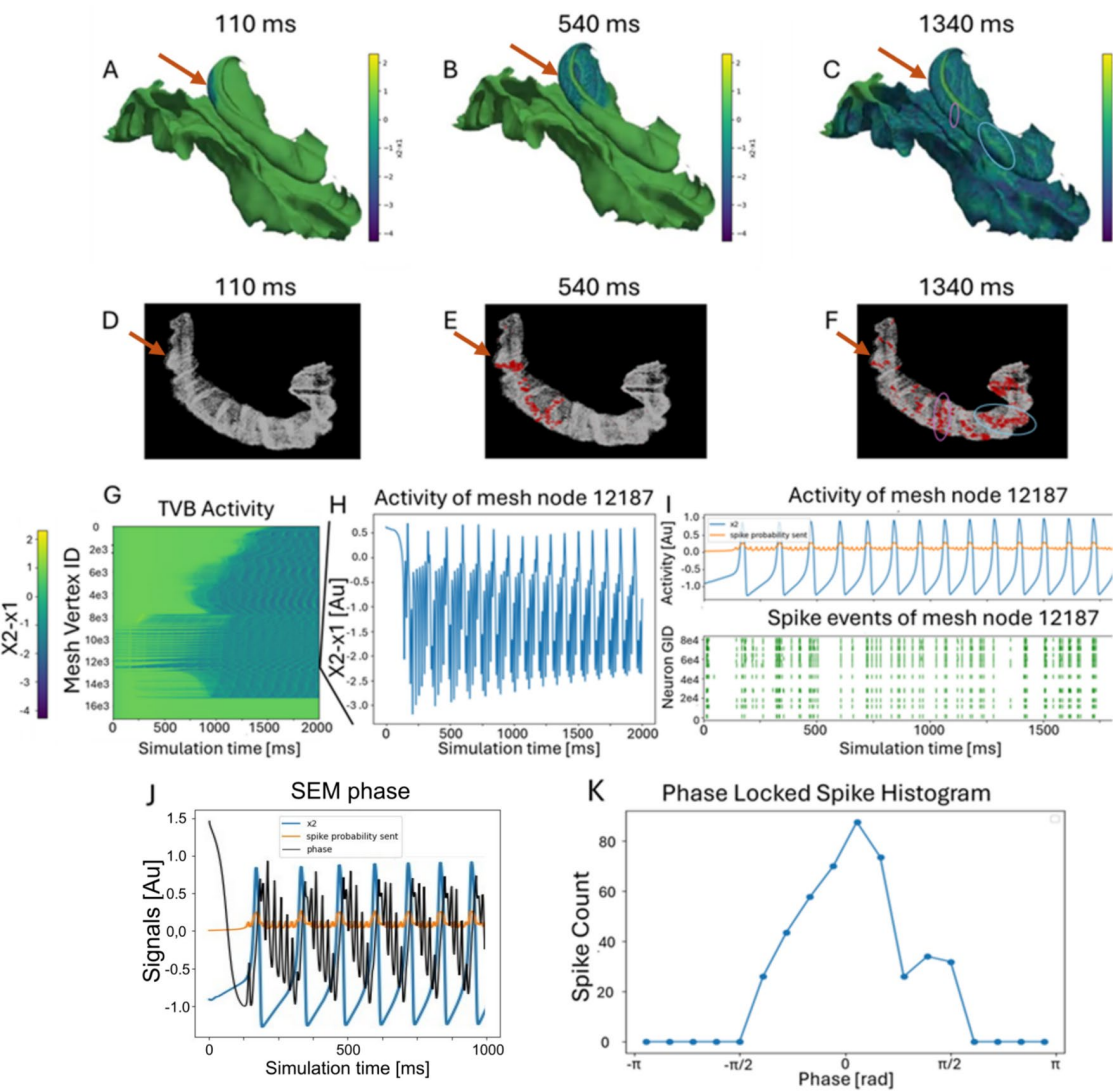
(**A**-**C**) **Temporal evolution of the TVB SEM**, showing the propagation of seizure-like events at three different time points. The onset site of the seizure comprises a subset of CA1 vertices and is pointed by the red arrow. (**D**-**F**) Temporal evolution of the NEST spiking neural network showing coherent activity (red dots) between TVB vertices and CA1 point neurons. Circled regions in C and F highlight the same regions in the two models. (**G**) Propagation of TVB node activity ($x_2-x_1$) as a time series across 16,636 vertices. (**H**) Timeseries of a single TVB node. (**I**) Comparison between TVB node $x_2$ driving signal (upper panel, blue), the activity converted spike probability sent to the NEST model (upper panel, yellow) and generated spike activity in the NEST model (lower panel). (**J**) The phase of the spike probability sent from TVB to NEST computed using the Hilbert transform and (**K**) average phase locked spike histogram across CA1 vertices and corresponding spiking subpopulations

## Results

### Preliminary results

The system was preliminarily validated by initializing both the TVB and NEST models in a steady state. The seizure-like event was then induced at the macroscale level (TVB), originating from the zone of onset of the seizure (a subset of CA1 regions) and triggering excitation that propagated to the microscale spiking neural network (SNN). To assess the validity of the co-simulation, we analyzed the relationship between SWE oscillations ($x_2$), rapid discharges ($x_1$), and synchronization within the SNN. At the macroscale level, the TVB model exhibited propagation of epileptic activity (Fig. 3A-C) from the hippocampus to adjacent areas of the temporal lobes. Both SWE events and fast discharges originated at seizure onset sites and spread to neighboring mesh nodes. At the microscale level, the NEST-based SNN showed corresponding burst-like synchronized activity (Fig. 3I-K), with spiking events propagating across the hippocampal structure (Fig. 3D-F). To further validate cross-scale coherence, we focused on a single CA1 vertex to illustrate seizure dynamics at both the macro- and microscale levels (Fig. 3H, I). To quantify synchronization between simulations, we computed phase-locked spike histograms. The phase of the TVB signals ($x_2$ - $x_1$) for each CA1 node was extracted using the Hilbert transform (Fig. 3J) and compared with spike counts from the corresponding NEST subpopulations. The resulting average phase-lock histograms (Fig. 3K) show that NEST spikes are concentrated around the phases with higher spike-generation probability, confirming tight phase synchronization between the two models. Finally, we replicated empirical patterns by comparing the simulated macroscale time series (analogous to local field potentials) with sorted neuronal spike trains recorded from microelectrodes during seizures, as reported in previous studies (Truccolo et al., 2014) (Fig. 3I).

To give an estimate of the required computational resources, 2000 ms were simulated in 20 minutes and 44 seconds on a desktop computer equipped with an Intel®Core™i9-13900K CPU (13th Gen, 16 cores / 32 threads) with 62 GB of RAM. The simulations were executed within a Debian-based environment running under the Windows Subsystem for Linux (WSL) on a Windows11 host operating system, using python 3.10.13 with MPI support via the mpi4py package version 4.1.0, a custom NEST package derived from version 3.8.0-post0.dev0 built through CMake and TVB library version 2.3. To achieve synchronization between the two simulators, the TVB waited an average time of 8.51± 10.24 ms for the NEST process to compute the simulation step.

### Discussion and conclusions

The proposed framework marks a significant advance in the integration of microscopically detailed spiking networks with whole-brain models, enabling the investigation of cross-scale dynamic interactions in human brain simulations. The conversion from seizure-like activity in the spatial epileptor model to spiking activity in the SNN effectively reproduced experimental pattern observed during human focal seizures, particularly those characterized by SWE complexes. This is largely due to the coupled subsystems of the Epileptor model, which separately represents rapid discharges and SWE dynamics, allowing for a more biologically grounded input to the spiking network. This co-simulation framework thus provides a powerful tool which allows for deeper insights regarding seizure dynamics across spatial and temporal scales. Future developments will target full-scale integration: expanding the TVB model to cover the entire brain surface and scaling the NEST model to include the entire CA1 region with approximately 40 billion synapses. Additionally, after a proper validation of the transfer function, bidirectional interaction will be enabled, allowing the spiking network to influence whole-brain dynamics. This will allow the study of how specific neuronal and synaptic parameters contribute to the initiation, propagation, and termination of seizures. Finally, future work will include a detailed analysis of meso- and macro-scale emergent dynamics across different frequency bands, as well as the long-range effects of local activity, further advancing our understanding of epilepsy and brain network behavior. On a larger scale, it can be envisaged the building of digital twin based on personal data and modified at the microcircuit level to explore pathological conditions and test personalized pharmacological strategies (see Supplementary materials). This approach has the potential to help us understand the mechanisms at the microscale level and ultimately contribute to the development of personalized medicine.

## Supplementary Information

Below is the link to the electronic supplementary material.

Supplementary file 1 (pdf 2286 KB)

## Data Availability

All codes developed to generate results and figures shown in the present manuscript will be made available upon request to the corresponding authors at the following link (https://github.com/NeuroLorenzo/Cosim_CA1_NEST_TVB_downscaled)

## References

[CR1] Gandolfi, D., Mapelli, J., Solinas, S. M. G., et al. (2023). Full-scale scaffold model of the human hippocampus ca1 area. *Nature Computational Science,**3*(3), 264–276. 10.1038/s43588-023-00417-2 38177882 10.1038/s43588-023-00417-2 PMC10766517

[CR2] Wang, H. E., Triebkorn, P., Breyton, M., Dollomaja, B., Lemarechal, J. D., Petkoski, S., Sorrentino, P., Depannemaecker, D., Hashemi, M., & Jirsa, V. K. (2024). Virtual brain twins: from basic neuroscience to clinical use. *National Science Review,**11*(5), 079. 10.1093/nsr/nwae079 10.1093/nsr/nwae079 PMC11065363 38698901

[CR3] Kusch, L., Diaz-Pier, S., Klijn, W., Sontheimer, K., Bernard, C., Morrison, A., & Jirsa, V. (2024). Multiscale co-simulation design pattern for neuroscience applications. *Frontiers in Neuroinformatics,**18*, 1156683. 10.3389/fninf.2024.1156683 38410682 10.3389/fninf.2024.1156683 PMC10895016

[CR4] Hater, T., Courson, J., Lu, H., Diaz-Pier, S., & Manos, T. (2025). *Arbor-TVB: A Novel Multi-Scale Co-Simulation Framework with a Case Study on Neural-Level Seizure Generation and Whole-Brain Propagation*. 10.48550/arXiv.2505.16861 10.3389/fncom.2025.1731161 PMC12907379 41704907

[CR5] Bragin, V., Dura-Bernal, S., Chen, J., Pai, R. K., Perdikis, D., Meier, J. M., Domide, L., Schirner, M., & Ritter, P. (2024). Interfacing biophysical circuits with whole-brain networks using the virtual brain and netpyne. *Journal of Computational Neuroscience,**52*(Suppl 1), 3–166. 10.1007/s10827-024-00871-5 39066873 10.1007/s10827-024-00871-5

[CR6] Gewaltig, M.-O., & Diesmann, M. (2007). Nest (neural simulation tool). *Scholarpedia,**2*(4), 1430. 10.4249/scholarpedia.1430

[CR7] Graber, S., Mitchell, J., Kurth, A.C., Terhorst, D., Skaar, J.-E.W., Schöfmann, C.M., Kunkel, S., Trensch, G., Haug, N., Mallett, D., Andriyovich, P.Y., Otazu Porter, X., Lee, A.Y., & Plesser, H.E. (n.d.) NEST 3.8. 10.5281/zenodo.12624784

[CR8] Sanz-Leon, P., Knock, S. A., Spiegler, A., & Jirsa, V. K. (2015). Mathematical framework for large-scale brain network modeling in the virtual brain. *NeuroImage,**111*, 385–430. 10.1016/j.neuroimage.2015.01.002 25592995 10.1016/j.neuroimage.2015.01.002

[CR9] Gandolfi, D., Mapelli, J., Solinas, S., De Schepper, R., Geminiani, A., Casellato, C., D’Angelo, E., & Migliore, M. (2022). A realistic morpho-anatomical connection strategy for modelling full-scale point-neuron microcircuits. *Scientific Reports,**12*(1), 13864. 10.1038/s41598-022-18024-y 35974119 10.1038/s41598-022-18024-y PMC9381785

[CR10] Hill, S., & Tononi, G. (2005). Modeling sleep and wakefulness in the thalamocortical system. *Journal of Neurophysiology,**93*(3), 1671–1698. 10.1152/jn.00915.2004 15537811 10.1152/jn.00915.2004

[CR11] Tsodyks, M., & Markram, H. (1997). The neural code between neocortical pyramidal neurons depends on neurotransmitter release probability. *Proceedings of the National Academy of Sciences,**94*(2), 719–723. 10.1073/pnas.94.2.719 10.1073/pnas.94.2.719 PMC19580 9012851

[CR12] Van Essen, D. C., Smith, S. M., Barch, D. M., Behrens, T. E. J., Yacoub, E., & Ugurbil, K. (2013). The wu-minn human connectome project: An overview. *NeuroImage,**80*, 62–79. 10.1016/j.neuroimage.2013.05.041 23684880 10.1016/j.neuroimage.2013.05.041 PMC3724347

[CR13] Glasser, M. F., Sotiropoulos, S. N., Wilson, J. A., et al. (2013). The minimal preprocessing pipelines for the human connectome project. *NeuroImage,**80*, 105–124. 10.1016/j.neuroimage.2013.04.127 23668970 10.1016/j.neuroimage.2013.04.127 PMC3720813

[CR14] Wang, H. E., Scholly, J., Triebkorn, P., Sip, V., Medina Villalon, S., Woodman, M. M., Le Troter, A., Guye, M., Bartolomei, F., & Jirsa, V. (2021). Vep atlas: An anatomic and functional human brain atlas dedicated to epilepsy patients. *Journal of Neuroscience Methods,**348*, 108983. 10.1016/j.jneumeth.2020.108983 33121983 10.1016/j.jneumeth.2020.108983

[CR15] Triebkorn, P., Wang, H.E., Woodman, M., Guye, M., Bartolomei, F., & Jirsa, V. (2025). Delay-constrained re-entry governs large-scale brain seizures and other network pathologies. 10.48550/arXiv.2508.04824

[CR16] Proix, T., Jirsa, V. K., Bartolomei, F., Guye, M., & Truccolo, W. (2018). Predicting the spatiotemporal diversity of seizure propagation and termination in human focal epilepsy. *Nature Communications,**9*, 1088. 10.1038/s41467-018-02973-y 29540685 10.1038/s41467-018-02973-y PMC5852068

[CR17] Jirsa, V. K., Stacey, W. C., Quilichini, P. P., Ivanov, A. I., & Bernard, C. (2014). On the nature of seizure dynamics. *Brain,**137*(8), 2210–2230. 10.1093/brain/awu133 24919973 10.1093/brain/awu133 PMC4107736

[CR18] DeKraker, J., Haast, R. A. M., Yousif, M. D., Karat, B., Lau, J. C., Köhler, S., & Khan, A. R. (2022). Automated hippocampal unfolding for morphometry and subfield segmentation with hippunfold. *ELife,**11*, 77945. 10.7554/eLife.77945 10.7554/eLife.77945 PMC9831605 36519725

[CR19] DeKraker, J., Palomero-Gallagher, N., Kedo, O., et al. (2023). Evaluation of surface-based hippocampal registration using ground-truth subfield definitions. *ELife,**12*, 88404. 10.7554/eLife.88404 10.7554/eLife.88404 PMC10642966 37956092

[CR20] Kurtzer, G. M., Sochat, V., & Bauer, M. W. (2017). Singularity: Scientific containers for mobility of compute. *PLOS ONE,**12*(5), 0177459. 10.1371/journal.pone.0177459 10.1371/journal.pone.0177459 PMC5426675 28494014

[CR21] Truccolo, W., Ahmed, O. J., Harrison, M. T., Eskandar, E. N., Cosgrove, G. R., Madsen, J. R., Blum, A. S., Potter, N. S., Hochberg, L. R., & Cash, S. S. (2014). Neuronal ensemble synchrony during human focal seizures. *Journal of Neuroscience,**34*(30), 9927–9944. 10.1523/JNEUROSCI.4567-13.2014 25057195 10.1523/JNEUROSCI.4567-13.2014 PMC4107409

