# Supplementary material for: Co-simulation framework combining a microscopically detailed point neuron model of the hippocampal CA1 region with the macroscopic high-resolution virtual brain model
Source: J Comput Neurosci. 2026 Feb 23;54(2):137–44. doi: 10.1007/s10827-026-00925-w (PMC13233651; doi:10.1007/s10827-026-00925-w)
Supplement: Supplementary file 1 — (pdf 2286 KB) [file 10827_2026_925_MOESM1_ESM.pdf]

SUPPLEMENTARY MATERIALS TO THE MANUSCRIPT: Co-simulation framework combining a microscopically detailed point neuron network model of the hippocampal CA1 region with the macroscopic high-resolution virtual brain model

Lorenzo Tartarini[1], Paul Triebkorn[2], Lionel Kusch[2], Sergio Solinas[3], Huifang Wang[2], Daniela Gandolfi[4], Viktor Jirsa[2], Jonathan Mapelli[1,4]

[1]Department of Biomedical, Metabolic and Neurosciences, University of Modena and Reggio Emilia, Modena, Italy.
[2]Aix Marseille Univ, INSERM, INS, Inst Neurosci Syst, Marseille, France.
[3]Department of Engineering, University of Sassari, Sassari, Italy.
[4]Department of Engineering "Enzo Ferrari", University of Modena and Reggio Emilia, Modena, Italy .

*Corresponding author(s). E-mail(s): jonathan.mapelli@unimore.it

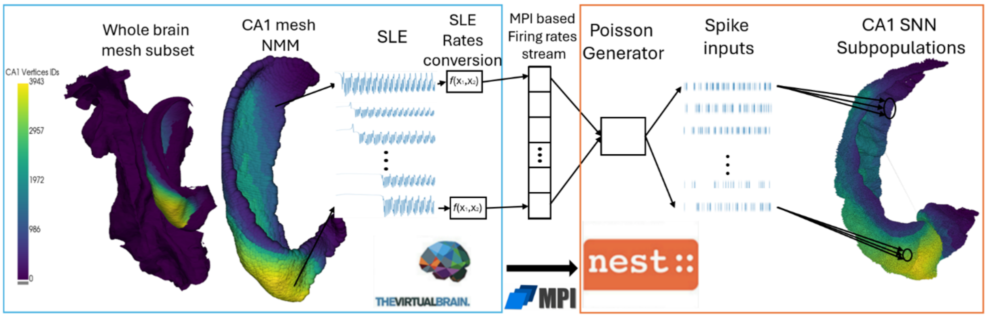

Fig. 1 Connection scheme. Connection scheme between the TVB macroscale with the NEST microscale simulators. Left. High resolution TVB region surrounding the CA1 region of the right hippocampus (zoomed in the middle). The CA1 region neural masses are represented based on spatial color code. Traces show Seizure Like Events (SLE) generated by the Spatial Epileptor Model (SEM) and propagating from an onset site. SLE traces are converted into firing rates through a transfer function to produce rate-based spike trains. Middle. The MPI protocol synchronized the computed firing rates with NEST simulator. Right. A custom designed Poisson generator converts firing rates into spike events. The resulting spike trains are delivered to the full scale CA1 model. Color code used to represent CA1 neurons in NEST is the same as in Left.

1 Code Availability

The code used to run the simulations described in this work, as well as the custom NEST version, is available upon request at https://github.com/NeuroLorenzo/Cosim_CA1_NEST_TVB_downscale.

2 Communication between TVB and NEST

The communication between TVB and NEST has been verified through the following procedures:

1. **Saving vectors**: Both the vectors of firing rates generated at each time step by TVB and NEST were saved
2. **Comparing vectors**: The consistency between vectors was ensured by checking the number of elements, the correct ordering, and data types (i.e., doubles).
3. **Verifying assignment**: the preservation of the neuron-to-mesh assignment was achieved by disabling all internal connections within NEST simulation, ensuring that the SNN activity was driven uniquely by the co-simulation input. Moreover, a few regions were randomly selected and stimulated with distinct activation rates (1 Hz, 10 Hz, 100 Hz). The resulting subpopulation activity in NEST was coherent with the input rates and consistent witht the predefined neuron-to-mesh mappings. The activated spiking regions accurately reflected both activity rates and the designed SNN-to-mesh point associations.
4. **Verifying the communication**: as shown in the main text, phase-locked histograms were generated to verify the correct communication between the two

simulators. Tests were performed in three conditions: (1) disabling NEST intraconnections and using x_2 as unique input drive, (2) using both x_1 and x_2 as input drive, and (3) enabling all the NEST intraconnections.

The designated devices correctly sampled the activity of the TVB nodes and transmitted the rate-coded information to the corresponding NEST subpopulation, respecting the predefined neuron-to-node assignments. All tests were also successfully performed using NEST's MPI-based parallelization, enabling the simulation to be distributed across multiple processes for improved performance

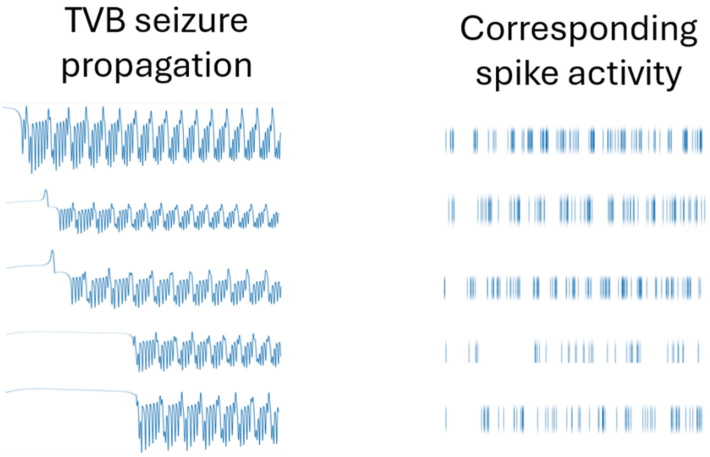

Fig. 2 Seizure propagation. Corresponding seizure propagation in TVB macroscale and NEST microscale simulators. Left. Spatial Epileptor Seizure-like activity (x_2-x_1) of TVB nodes progressively further from the onset site. Right. NEST spiking activity of neurons sampled from the corresponding network subpopulation, driven by TVB activity through custom designed rate coding.

3 Downsampling the CA1 point neuron model

The full-scale CA1 network model has been downscaled by randomly sampling GID from the full list of neurons. Consequently, only connections among the selected neurons were preserved to build the downscaled network. The downscaling procedure has been verified by comparing salient network properties, like the neuronal density distribution (Fig. SM3 A-C), the indegree and outdegree distribution (Fig. SM3 D-E) and the distribution of the connection lengths (Fig. SM3 F). It should be noted that, despite the overall distribution of the connection length with two peaks at the same length being preserved, a difference in the relative density of the two peaks can be observed. This is probably due to inhibitory connections, which do not show shorter lengths because of reduced cell density after the downsampling procedure (data not shown).

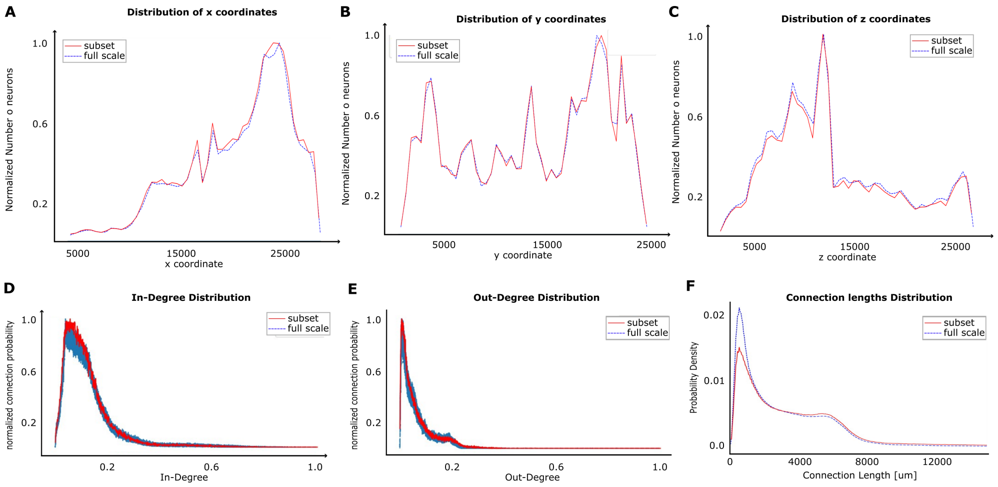

Fig. 3 Structural properties of the downsampled network. Comparison between full-scale hippocampal CA1 network and its down sampled version. (A-C) Displacement comparison across xyz axis. (D-E) Indegree and Outdegree probability comparison, normalized by maximum probability value. (F) Connection lengths distribution comparison, computed as Euclidian distance between connected neurons.

4 Microscopical spatio-temporal scale network alterations

Using the proposed framework, the effects of the detailed CA1 network activity driven by epileptic neighboring regions, as simulated using TVB spatial epileptor model, can be investigated. Namely, the analysis of the effects of the changes in excitatory and inhibitory synaptic weights resulted in altered activity (Fig. SM4) which can be estimated by spike counting. The CA1 generated a total of 4'504'888 spikes in control condition; 4'019'548 spikes with 10% excitation (89% of the control spike counts; all weights of excitatory connections were decreased to 10% of the control value); 3'957'702 spikes with 500% inhibition (88% of the control spike counts; all weights of inhibitory connections were increased to 5 times the control value); 3'861'275 spikes with 1000% inhibition (86% of the control spike counts); 3'818'978 spikes with 2000% inhibition (85% of the control spike counts); 5'375'658 spikes without inhibition (119% of the control spike counts). Furthermore, according to the ILAE (International League Against Epilepsy) definitions, the sclerosis and gliosis of the hippocampus can be involved in temporal lobe epilepsy. In the attempt to mimic these conditions, we have modeled the structural alterations of the CA1. In the first case (only sclerosis - SCL1) all connections within a radius of 6 mm centered on the tip of the uncus region have been removed while external inputs were preserved. In the second case, a more severe condition including gliosis, the internal connections were removed as well as external inputs from TVB (SCL2) [1]. In case of SCL1 the total emitted spikes were 3'798'265 (84% of the control spike counts); in SCL2 the total emitted spikes were 3'487'480 (77% of the control spike counts).

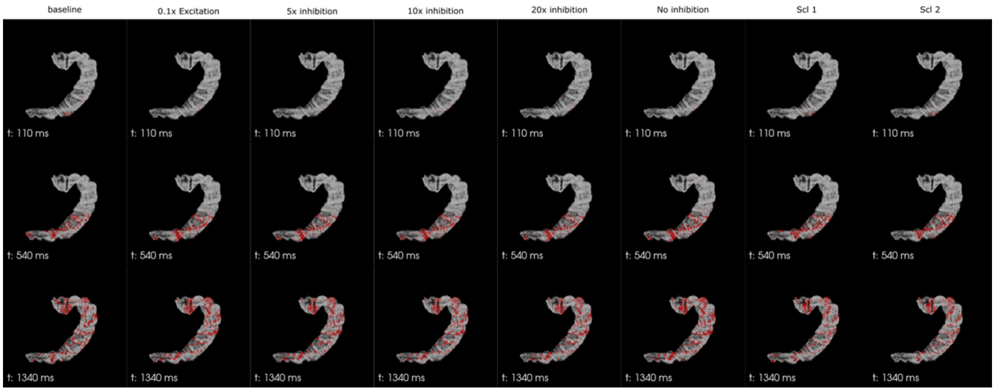

Fig. 4 **Inhibition Sweep.** Comparison of the TVB-driven NEST activity under varying excitatory and inhibitory connection weights. From left to right: Control CA1 activity at three timeframes represented by different rows (t=110ms, t= 540ms, t= 1340ms); activity with excitatory synaptic weights reduced to 10% of the control condition; activity with inhibitory weights increased to 500%, 1000%, 2000%; and activity with inhibitory connections removed. Hippocampal sclerosis condition with (SCL1) and without (SCL2) external inputs from TVB.

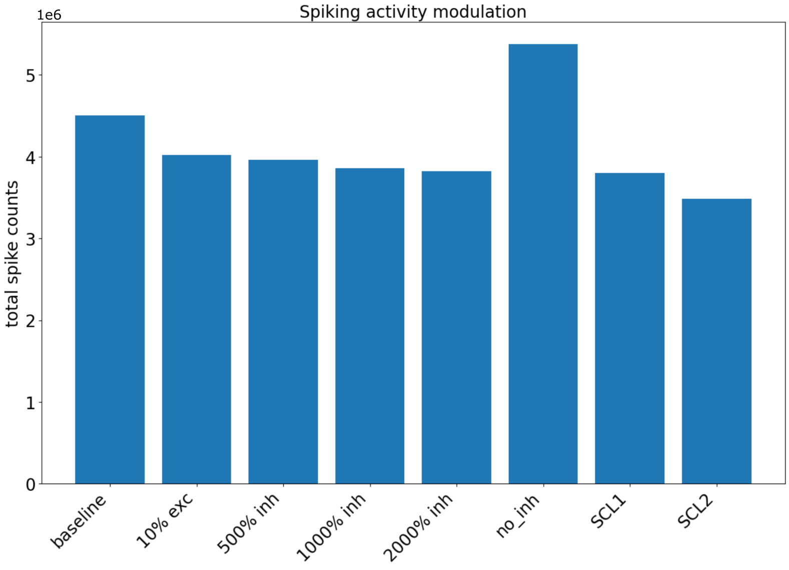

Fig. 5 **Inhibition Sweep Spike counts.** Histogram of total number of generated spike in each SNN configuration.

5 Spatial Epileptor Model dynamics

The parameters of the spatial epileptor model were chosen to reproduce biologically plausible dynamics of fast discharges state (x_1) and spike-and-wave complexes state (x_2). Seizure activity propagates from the onset region throughout the mesh with

oscillations in the gamma and theta band for the x_1 and x_2 state respectively (Fig. SM5).

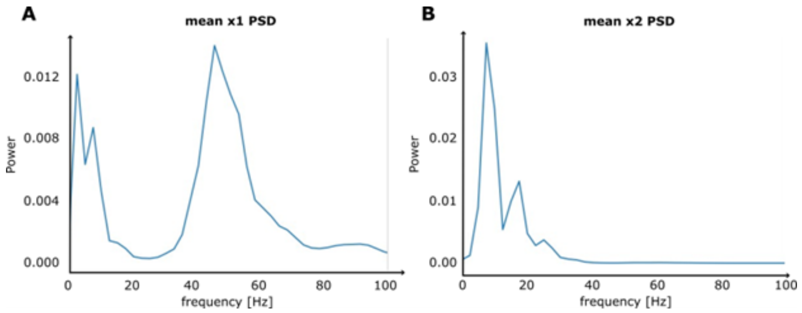

Fig. 6 Spatial Epileptor Spectrum. Power spectral density (PSD) analysis of the two main components of the TVB activity: fast discharges state (x_1) and spike-and-wave complexes state (x_2). The PSD was computed for each TVB node using the Welch method. (A) Average PSD of x_1 across all TVB nodes. (B) Average PSD of x_2 across all TVB nodes.

6 Model Equations

6.1 Spatial Epileptor Model's equations

The Spatial Epileptor Model consists of the following 6 states-equations [2]:

$$\dot{x_1} = tt(y_1 - f_1(x_1, x_2, z) - z + I_{ext} + loc_{11} + c_{pop}) \tag{1}$$

$$\dot{y_1} = tt(y_0 - 5x_1^2 - y_1) \tag{2}$$

$$\dot{z} = tt(\frac{1}{\tau_0}(4((x_1 - x_0) - z + f_3(z)) \tag{3}$$

$$\dot{x_2} = tt(-y_2 + x_2 - x_2^3 + I_{ext2} + 2g - 0.3(z - 3.5) + loc_{22} \tag{4}$$

$$\dot{y_2} = tt(\frac{1}{\tau_2}(-y_2 + f_2(x_2, y_2)) \tag{5}$$

$$\dot{g} = tt(-0.01g + 0.003x_1 + 0.01loc_{12}) \tag{6}$$

$$f_1(x_1, x_2, z) = \begin{cases} x_1^3 - 3x_1^2, & \text{for } x_1 < 0 \\ (x_2 - 0.6(z-4)^2)x_1, & \text{for } x_1 \geq 0 \end{cases} \tag{7}$$

$$f_2(x_2) = \begin{cases} 0, & \text{for } x_1 < -0.25 \\ 6(x_2 + 0.25), & \text{for } x_2 \geq -0.25 \end{cases} \tag{8}$$

$$f_3(z) = \begin{cases} -0.1z^7, & \text{for } z < 0 \\ 0, & \text{for } z \geq 0 \end{cases} \tag{9}$$

where tt is the temporal scaling of the equations

6.2 Local coupling functions' equations

,

$$loc_{11} = \gamma_{11} w_1 \cdot \mathrm{H}(x_1 - \theta_{11}) \tag{10}$$
$$loc_{12} = \gamma_{12} w_{12} \cdot \mathrm{H}(x_1 - \theta_{12}) \tag{11}$$
$$loc_{22} = \gamma_{22} w_{22} \cdot \mathrm{H}(x_2 - \theta_{22}) \tag{12}$$

use a Heaviside coupling function $\mathrm{H}(\cdot)$ and a spatial convolution defined as

$$w \cdot H(x, \theta) := \int_{-\infty}^{+\infty} w(y) H(x(t) - \theta) \mathrm{d}y \tag{13}$$

with w(y) representing the connection strength between sites separated by a geodesic distance y and γ_{ij} are coupling scaling factors.

6.3 Global coupling functions' equations

The global coupling function

$$c_{pop} = \gamma_{glob} G_s \tag{14}$$

where

$$G_{s_i} = \sum_j w_{ij}^G H(x_{1,j}(t - d_{ij}) - \theta_G) \tag{15}$$

is the global connectivity input received by node i, computed as weighted sum of delayed nodes activity passed through the Heaviside activation function with threshold θ_G. w_{ij}^G denotes the structural connectivity weight between nodes i and j. The delays d_{ij} are computed as length of the streamlines connecting the nodes, divided by a fixed conduction speed of 3.6 m/s. x_1, y_1 represents rapid discharge states, x_2, y_2 spike and wave events states, z permittivity state that drives the system between ictal and non-ictal periods and g integral coupling function.

Regarding the NEST models, the Hill Tononi Neuron model equations correspond to those implemented natively in NEST [3, 4] https://nest-simulator.readthedocs.io/en/latest/model_details/HillTononiModels.html, which are a slightly modified version of the neuron model originally described in [5]. The Tsodyks-Markram synapse model equations are likewise those provided by NEST https://nest-simulator.readthedocs.io/en/latest/models/tsodyks_synapse.html, implementing synaptic short-term depression and short-term facilitation as described in [6] . Synapse and neuron parameters have been set according to [7]

Table 1 Software packages and versions. Here is a full list of the NEST, TVB and python packages installed to run co-simulations.

Package	Version	Package	Version
Addict	2.4.0	Numba	0.58.1
antlr4-python3-runtime	4.13.2	numexpr	2.11.0
astropy	6.1.7	numpy	1.23.5
astropy-iers-data	0.2025.7.21.0.41.39	odetoolbox	2.5.9
asttokens	3.0.0	open3d	0.19.0
attrs	25.3.0	packaging	25.0
autopep8	2.3.2	pandas	2.3.1
blinker	1.9.0	parso	0.8.4
certifi	2025.7.14	pexpect	4.9.0
charset-normalizer	3.4.2	pillow	11.3.0
click	8.2.1	pip	25.1.1
colour	0.1.5	platformdirs	4.3.8
comm	0.2.2	plotly	6.2.0
ConfigArgParse	1.7.1	pooch	1.8.2
Contourpy	1.2.1	prompt_toolkit	3.0.51
cycler	0.12.1	ptyprocess	0.7.0
Cython	3.0.12	pure_eval	0.2.3
Dash	3.1.1	pycodestyle	2.14.0
Decorator	5.2.1	pyerfa	2.0.1.5
Errr	1.2.0	Pygments	2.19.2
exceptiongroup	1.3.0	pyparsing	3.2.3
executing	2.2.0	pyquaternion	0.9.9
fastjsonschema	2.21.1	python-dateutil	2.9.0.post0
Flask	3.1.1	pytz	2025.2
Fonttools	4.59.0	pyvista	0.45.3
h5py	3.7.0	PyYAML	6.0.2
idna	3.10	referencing	0.36.2
imageio-ffmpeg	0.6.0	requests	2.32.4
importlib_metadata	8.7.0	retrying	1.4.1
importlib_resources	6.5.2	rpds-py	0.26.0
ipycanvas	0.13.3	rtree	1.4.0
ipyevents	2.0.2	scikit-learn	1.7.1
ipython	8.37.0	scipy	1.10.1
ipyvtklink	0.2.2	scooby	0.10.1
ipywidgets	8.1.7	setuptools	65.5.0
itsdangerous	2.2.0	six	1.17.0
jedi	0.19.2	stack-data	0.6.3
Jinja2	3.1.6	sympy	1.14.0
Joblib	1.5.1	threadpoolctl	3.6.0
jsonschema	4.25.0	tomli	2.2.1
jsonschema-specifications	2025.4.1	tqdm	4.67.1
jupyter_core	5.8.1	traitlets	5.14.3
jupyterlab_widgets	3.0.15	trimesh	4.7.1
kiwisolver	1.4.8	tvb-contrib	2.3
llvmlite	0.41.1	tvb-data	2.0
Mako	1.3.10	tvb-gdist	2.0.0
MarkupSafe	3.0.2	tvb-library	2.3
matplotlib	3.8.4	typing	3.7.4.3
matplotlib-inline	0.1.7	typing_extensions	4.14.1
mpi4py	4.1.0	tzdata	2025.2
mpmath	1.3.0	urllib3	2.5.0
narwhals	1.48.0	vtk	9.4.2
nbformat	5.10.4	wcwidth	0.2.13
NEST	3.8.0-post0.dev0*	Werkzeug	3.1.3
nest-asyncio	1.6.0	wheel	0.45.1
NESTML	8.0.2	widgetsnbextension	4.0.14
networkx	3.4.2	xarray	2025.6.1
nibabel	5.3.2	zipp	3.23.0
nose	1.3.7		

* Custom version built starting from the c++ codebase referenced, with added devices and models.

References

[1] Blümcke, I., Thom, M., Aronica, E., Armstrong, D.D., Bartolomei, F., Bernasconi, A., Bernasconi, N., Bien, C.G., Cendes, F., Coras, R., Cross, J.H., Jacques, T.S., Kahane, P., Mathern, G.W., Miyata, H., Moshé, S.L., Oz, B., Ozkara, C., Perucca, E., Sisodiya, S., Wiebe, S., Spreafico, R.: International consensus classification of hippocampal sclerosis in temporal lobe epilepsy: A task force report from the ilae commission on diagnostic methods. Epilepsia **54**(7), 1315–1329 (2013) https://doi.org/10.1111/epi.12220 https://onlinelibrary.wiley.com/doi/pdf/10.1111/epi.12220

[2] Proix, T., Jirsa, V.K., Bartolomei, F., Guye, M., Truccolo, W.: Predicting the spatiotemporal diversity of seizure propagation and termination in human focal epilepsy. Nature Communications **9**, 1088 (2018) https://doi.org/10.1038/s41467-018-02973-y

[3] Gewaltig, M.-O., Diesmann, M.: Nest (neural simulation tool). Scholarpedia **2**(4), 1430 (2007) https://doi.org/10.4249/scholarpedia.1430

[4] Graber, S., Mitchell, J., Kurth, A.C., Terhorst, D., Skaar, J.-E.W., Schöfmann, C.M., Kunkel, S., Trensch, G., Haug, N., Mallett, D., Andriyovich, P.Y., Otazu Porter, X., Lee, A.Y., Plesser, H.E.: NEST 3.8. https://doi.org/10.5281/zenodo.12624784 . https://doi.org/10.5281/zenodo.12624784

[5] Hill, S., Tononi, G.: Modeling sleep and wakefulness in the thalamocortical system. Journal of Neurophysiology **93**(3), 1671–1698 (2005) https://doi.org/10.1152/jn.00915.2004

[6] Tsodyks, M., Markram, H.: The neural code between neocortical pyramidal neurons depends on neurotransmitter release probability. Proceedings of the National Academy of Sciences **94**(2), 719–723 (1997) https://doi.org/10.1073/pnas.94.2.719

[7] Gandolfi, D., Mapelli, J., Solinas, S.M.G., *et al.*: Full-scale scaffold model of the human hippocampus ca1 area. Nature Computational Science **3**(3), 264–276 (2023) https://doi.org/10.1038/s43588-023-00417-2
